# Effects of obesity on short-term mortality in patients with acute heart failure under different nutritional status

**DOI:** 10.1186/s12872-023-03206-x

**Published:** 2023-04-29

**Authors:** Linlin Liu, Jun Qian, Yuanyuan Li, Ye Ni, Ya Zhao, Lin Che

**Affiliations:** 1 Department of Cardiology, Shanghai Tongji Hospital, Tongji University School of Medicine, Shanghai, 200092 China; 2 Department of Cardiology, Kong Jiang Hospital Of Yangpu District, Shanghai, 200093 China

**Keywords:** Acute heart failure, Obesity paradox, Nutritional status, Mortality

## Abstract

**Background:**

Increased body mass index (BMI) is associated with better survival in patients with acute heart failure (AHF), which is a paradoxical phenomenon. However, it is unclear whether different nutritional status affects this association.

**Methods:**

1325 patients with AHF from the Medical Information Mart for Intensive Care III database were retrospectively included. Nutritional status was assessed by serum albumin (SA) and prognostic nutritional index (PNI). Patients were divided into High-SA (≥ 3.5 g/dL) and Low-SA groups (< 3.5 g/dL), and they also were divided into High-PNI (≥ 38) and Low-PNI groups (< 38). Propensity-score matching (PSM) was used to control for the effect of baseline confounding factors, multifactor regression model was adopted to assess the association of nutritional status, BMI, and outcomes in AHF patients.

**Results:**

Of the 1325 patients (mean age 72.4 ± 13.1 years), 52.1% (n = 690) were male, 13.1% (n = 173) died in hospital and 23.5% (n = 311) died within 90 days. Before PSM, after adjusting for potential confounders, in the High-SA population, compared with the under/normal BMI group, overweight and obesity were negatively correlated with 90-day mortality, with adjusted hazard ratios (HR) of 0.47, 95% confidence interval (CI) (0.30–0.74), P = 0.001; HR 0.45, 95%CI (0.28–0.72), P = 0.001, respectively. However, this correlation was much attenuated in the Low-SA group (overweight BMI: HR 1.06, 95%CI 0.75–1.50, P = 0.744; obese BMI: HR 0.86, 95%CI 0.59–1.24, P = 0.413). After PSM, those who were overweight or obese in the High-SA group had a 50–58% reduction in 90-day risk of death, while the protective effect disappeared in the Low-SA group (HR 1.09, 95% CI 0.70–1.71; HR 1.02, 95%CI 0.66 − 0.59). Similarly, results were similar in analyses using PNI as a nutritional assessment criterion.

**Conclusion:**

Overweight or Obesity was associated with lower short-term mortality in well-nourished AHF patients, whereas this association was significantly attenuated or even disappeared in malnourished patients. Therefore, further research is needed for weight loss recommendations for malnourished obese patients with AHF.

**Supplementary Information:**

The online version contains supplementary material available at 10.1186/s12872-023-03206-x.

## Introduction

Acute heart failure (AHF) is a clinical syndrome of a sharp decline in cardiac function, which is caused by various reasons such as myocardial injury and cardiac overload. High prevalence, high disability, and high fatality rate seriously affect patients’ quality of life [1–3]. Therefore, understanding the prognostic factors of HF may play a vital role in assisting clinicians to identify high-risk patients and implement interventions for related risk factors as soon as possible.

With the development of society and changes in human lifestyles, the prevalence of Obesity has increased exponentially, and it has become an increasingly social severe public health problem [4]. As is known, Obesity can lead to severe cardiovascular system damage [5], especially for the occurrence and development of HF [6]. Paradoxically, once HF is diagnosed, obese patients have a distinct survival advantage over leaner individuals, the so-called “obesity paradox” [7]. Up to now, there have been various explanations to elucidate this phenomenon. Whether this counterintuitive epidemiological association between survival outcomes and traditional risk factors is a real phenomenon or the result of reverse causality or collider stratification bias remains unclear [8]. Nevertheless, this phenomenon has been repeatedly demonstrated in a variety of diseases, including stroke [9], peripheral arterial disease [10], acute coronary syndrome (ACS) [11], and sepsis [12].

In addition, many countries currently face the double burden of malnutrition and Obesity. Malnutrition is prevalent in HF and is related to poor prognosis [13]. A recent meta-analysis indicated that the prevalence of malnutrition risk in HF was 16–90%, as high as 75–90% in patients with AHF, and confirmed that malnutrition was significantly associated with poor prognosis and high mortality [14]. Various nutritional screening tools such as serum albumin (SA), serum preprotein, prognostic nutritional index (PNI) [15], nutritional risk screening (NRS), and geriatric nutritional risk Index (GNRI) were adopted to assess nutritional risk in patients with HF [13, 16], but there is no consensus on which tool is more accurate. In this study, SA and PNI were used as alternative markers of nutritional status. Previous studies have mainly used a single BMI or nutritional index to predict the clinical outcomes of AHF. Whether the contradiction between Obesity and the prognosis of AHF patients still exists under different nutritional statuses is rarely reported. Therefore, this study aimed to investigate the effect of Obesity on all-cause mortality of AHF under different nutritional statuses.

## Methods

### Database introduction

We conducted a retrospective study collecting data from the Medical Information Mart for Intensive Care III (MIMIC-III) database [17]. The database contains clinical information of over 40,000 patients admitted to the Intensive Care Unit (ICU) of Beth Israel Deaconess Medical Center (BIDMC) in Boston, Massachusetts between 2001 and 2012(14). In order to facilitate research by researchers all over the world, the database hid patient information and passed the approval of the Massachusetts Institute of Technology (MIT, Cambridge, MA, USA) Institutional Review Board (IRB), and BIDMC, eliminating the need for informed consent Requirement, but only if the researcher needs to complete an online training course to access the database.

### Study population and definition

We used the International Classification of Diseases (ICD) -9 diagnosis codes to extract all patients (age ≥ 18 years) diagnosed with AHF from the MIMIC-III database (ICD-9 codes: 428.21, 428.23, 428.31, 428.33, 428.41, 428.43). Exclusion criteria were as follows: (1) patients without height, weight, and SA data after admission; (2) patients with ACS, sepsis, malignancy, severe liver cirrhosis (Child-Pugh score B or C), chronic renal dysfunction (stage IV or V); (3) patients in pregnancy, childbirth or puerperium; (4) ICU stay ≤ 48 h; (5) patients with repeated admissions will be deleted, and only the information of the first admission will be retained. The flow chart was shown in Additional File Fig. 1.

Nutritional status was defined based on the SA concentration of the candidates (Low-SA, < 3.5 g/dL [malnutrition]; High-SA, ≥ 3.5 g/dL [well-nutrition]) [18]. PNI, another indicator used to assess nutritional status, is calculated according to the formula: 10×SA (g/dL) + 5×total lymphocyte count×10^9^ /L. A value ≥ 38 (High-PNI) is considered well-nourished, and a value < 38 is considered malnourished [19]. BMI is calculated as weight (kg) divided by height (meters) squared. Based on the classification standards of the World Health Organization (WHO) BMI, patients were divided into four groups: underweight (< 18.5 kg/m^2^), normal weight (18.5 to < 25 kg/m^2^), overweight (25–30 kg/m^2^), obesity (> 30 kg/m^2^). Whereas, considering that the number of underweight patients was only 27, patients were divided into three groups: under/normal BMI (< 25 kg/m^2^), overweight BMI (25–30 kg/m^2^), and obese BMI (> 30 kg/m^2^).

### Data extraction and processing

The structured query language (SQL) was used to collect clinical information from the database, including age, gender, smoking status, physical examination indicators, Sequential Organ Failure Assessment (SOFA) score, hospital and ICU length of stay (LOS), survival status, laboratory test indicators, comorbidities, drug use, and adjuvant treatment measures (continuous renal replacement therapy [CRRT], use of ventilator). Physical examination indicators include: height, weight, and heart rate(HR); Laboratory tests were extracted the first measured values within 24 h after admission to the ICU, including white blood cell count (WBC), hemoglobin (HB), anion gap (AG), lymphocyte count, creatinine, SA, glucose, N-terminal probrain natriuretic peptide (NT-proBNP); Complications such as hypertension, diabetes, chronic obstructive pulmonary disease (COPD), prior myocardial infarction (MI), atrial fibrillation (AF), hyperlipidemia and cardiogenic shock; Use of admission medications such as β-blockers, diuretics, digoxin, angiotensin-converting enzyme inhibitors (ACEIs).

In addition, more than 20% of patients did not have NT-proBNP data, which was converted into dummy variables for analysis so as to avoid possible bias caused by directly filling in missing values. It was worth noting that in patients with NT-proBNP values, only 15 patients with NT-proBNP values less than 400pg/mL. Therefore, the median was used here as the cutoff value for dummy variable conversion, which could not adequately distinguish the severity of the AHF. Furthermore, the birth date of patients over 89 years old has been moved to conceal their actual age, causing these patients to appear in the database over 300 years old. In fact, the median age of these patients is 91.4 years, so we modify the age of these patients to 91.4 years.

### Outcomes

The primary outcome was 90-day mortality, and the secondary outcomes were in-hospital mortality, hospital LOS, and ICU LOS. Survival time was defined as the day from admission to death. The death information was extracted from the social security death index.

### Statistical analysis

Based on literature reviewing, the sample size calculation for this study was based on 90-day all-cause mortality outcome events. Assuming a 90-day all-cause mortality rate of 18% in the malnourished group [20–22], and an approximately 10% reduction in the well-nourished group. Using a two-tailed alpha error of 0.05, the sample size of 394 patients in each group was estimated to provide 90% statistical power. Considering the 20% lost to follow-up, each group would require an estimated minimum of 493 patients (i.e., 986 patients in total) to achieve the expected effect. Hence, our sample size of 1181 patients was able to detect the significant difference with sufficient power.

All analyses were operated using software Stata, version 14.0 and R version 4.0.5 (R Foundation for Statistical Computing, Vienna, Austria). Based on the distribution and variance of data, continuous variables were expressed as mean ± standard deviation (SD) or median (interquartile range, IQR), and compared using Student’s t-test or Mann–Whitney U-test. Categorical variables were represented by composition ratio and compared applying χ2-test or Fisher’s exact test. To reduce the interference of confounding factors on outcome events, the nearest neighbor matching method was selected to perform 1:1 propensity-score matching (PSM) between the High-SA and Low-SA groups, and the caliper value was set to 0.02. The Kaplan-Meier (KM) curves were used to analyze cumulative survival in each subgroup, and the log-rank test was used to compare differences between groups. Cox regression models were used to analyze associations between BMI, nutritional status and outcomes, expressed as hazard ratios (HR) and relative 95% confidence intervals (CI). In order to avoid the interference of potential confounding factors, age, gender, and all variables with *P* < 0.05 in Table 1 were picked into the final regression model. In addition, considering that the single SA concentration may be affected by non-nutritional factors such as water and sodium retention, inflammation, etc., we also performed a series of sensitivity analyses on patients with PNI data. When the two-tailed P < 0.050, a statistically significant difference was set.

**Table 1:** Baseline characteristics of the study population grouped by SA before and after propensity-score matching

Variables	Before PSM				After PSM				
	High-SA (n = 645)	Low-SA (n = 680)	P value		High-SA (n = 528)			Low-SA (n = 528)	P value
**Demographics**									
Age(years)	72.0 ± 13.0	72.7 ± 13.3		0.329	72.2 ± 12.6		72.0 ± 13.6		0.788
Male, n (%)	359(55.7)	331(48.7)		0.011^*^	275(52.1)		274(51.9)		0.951
Smoking, n (%)	190(29.5)	176(25.9)		0.146	149(28.2)		140(26.5)		0.534
BMI (kg/m^2^)	28.2(24.5–32.9)	27.7(23.7–32.4)		0.162	28.2(24.5–32.7)		28.0(24.3–33.1)		0.990
HR (b.p.m)	87(74–100)	89(78–103)		0.002^**^	87(75–102)		87(76–101)		0.878
Hypertension, n (%)	303(47.0)	270(39.7)		0.008^**^	233(44.1)		226(42.8)		0.664
Prior MI, n (%)	86(13.3)	90(13.2)		0.958	65(12.3)		73(13.8)		0.465
Hyperlipidemia, n (%)	209(32.4)	186(27.4)		0.045^*^	152(28.8)		161(30.5)		0.544
COPD, n (%)	42(6.5)	45(6.6)		0.938	34(6.4)		32(6.1)		0.799
AF, n (%)	353(54.7)	356(52.4)		0.386	288(54.5)		273(51.7)		0.355
Diabetes, n (%)	251(38.9)	280(41.2)		0.401	202(38.3)		229(43.4)		0.118
Cardiogenic shock, n (%)	62(9.6)	97(14.3)		0.009^**^	56(10.6)		53(10.0)		0.762
Digoxin, n (%)	99(15.3)	108(15.9)		0.789	83(15.7)		83(15.7)		1.000
β-blockers, n (%)	544(84.3)	555(81.6)		0.188	446(84.5)		437(82.8)		0.454
ACEI, n (%)	284(44.0)	267(39.3)		0.079	223(42.2)		225(42.6)		0.901
Diuretic, n (%)	597(92.6)	638(93.8)		0.360	489(92.6)		500(94.7)		0.165
WBC, 10^9^/L	10.5(7.7–14.4)	11.5(8.3–16.1)		< 0.001^***^	11.2(8.2–15.1)		10.6(8.0-14.4)		0.254
HB, mg/dL	10.4(8.9–12.2)	9.9(8.8–11.4)		< 0.001^***^	10.1(8.8–11.7)		10.1(9.0-11.7)		0.453
AG, mmol/L	14(11–16)	14(12–17)		0.059	14(11–17)		14(12–16)		0.982
Creatinine, mg/dL	1.2(0.9–1.7)	1.3(0.9-2.0)		0.002^**^	1.2(0.9–1.8)		1.3(0.9–1.9)		0.263
Glucose, mg/dL	122(102–152)	122(99–156)		0.748	122(101–153)		124(101–158)		0.338
NT-proBNP, n (%)				0.009					0.259
< 6217 pg/mL	153(23.7)	134(19.7)			115(21.8)		108(20.5)		
≥ 6217pg/mL	102(15.8)	150(22.1)			91(17.2)		112(21.2)		
No test	390(60.5)	396(58.2)			322(61.0)		308(58.3)		
SOFA scores	4.0(3.0–7.0)	5.0(3.0–8.0)		< 0.001^***^	5.0(3.0–7.0)		5.0(3.0–7.0)		0.566
CRRT, n (%)	20(3.1)	45(6.6)		0.003^**^	20(3.8)		16(3.0)		0.498
Assisted ventilation, n (%)	349(54.1)	386(56.8)		0.331	282(53.4)		289(54.7)		0.666
**Outcomes**									
Hospital mortality, n (%)	61(9.5)	112(16.5)		< 0.001^***^	53(10.0)		67(12.7)		0.175
90-day mortality, n (%)	116(18.0)	195(28.7)		< 0.001^***^	97(18.4)		132(25.0)		0.009^**^
Hospital LOS, days	10.3(7.0-15.4)	12.7(8.3–18.9)		< 0.001^***^	10.7(7.0-15.8)		11.6(8.1–18.2)		0.002^**^
ICU LOS, days	4.2(3.0–7.0)	5.1(3.4–9.5)		< 0.001^***^	4.2(3.0-7.1)		4.9(3.2–8.1)		0.001^**^

BMI, body mass index; HR, heart rate; MI, myocardial infarction; AF, Atrial fibrillation; COPD, chronic obstructive pulmonary disease; ACEI, angiotensin-converting enzyme inhibitors; WBC, white blood cell count; HB, Hemoglobin; SA, serum albumin; AG, anion gap; NT-proBNP, N-terminal probrain natriuretic peptide; SOFA, sequential organ failure assessment; CRRT, continuous renal replacement therapy; ICU, Intensive Care Unit; LOS, length of stay; PSM, propensity-score matching; ^*^P < 0.05; ^**^P < 0.01; ^***^P < 0.001

## Results

### Clinical baseline characteristics of patients

The baseline characteristics of patients in the High- and Low-SA groups before and after PSM are presented in Table 1, the distribution of patients after PSM is shown in Additional File Fig. 2. Before PSM, among the 1325 patients enrolled (mean age 72.4 years, male 52.1%). 645 patients (48.7%) were in the High-SA (well-nourished) group and 680 (51.3%) in the Low-SA group. Compared with the High-SA group, patients in the Low-SA group had lower body weight, lower BMI, faster HR, a higher proportion of CRRT use (6.6% vs. 3.1%) and greater prevalence of cardiogenic shock (14.3% vs. 9.6%). In addition, they had higher WBC levels, creatinine levels, NT-proBNP levels and SOFA scores, while hemoglobin levels and admission blood glucose were lower. Hypertension (44.3%) and hyperlipidemia (34.7%) were more common in patients with SA ≥ 3.5 g/dL. Of note, compared with the High-SA group, the Low-SA group showed poorer clinical outcomes such as higher in-hospital and 90-day mortality (16.5% vs. 9.5%, 28.7% vs. 18.0%, all P < 0.001), longer hospital stay and ICU stay (12.7[8.3–18.9] vs. 10.3[7.0-15.4], 5.1[3.4–9.5] vs. 4.2[3.0–7.0], all *P* < 0.001). After PSM, there were no significant differences in all baseline variables between the two groups for 528 pairs of patients, with those in the Low-SA group having worse outcomes.

**Fig. 1:**
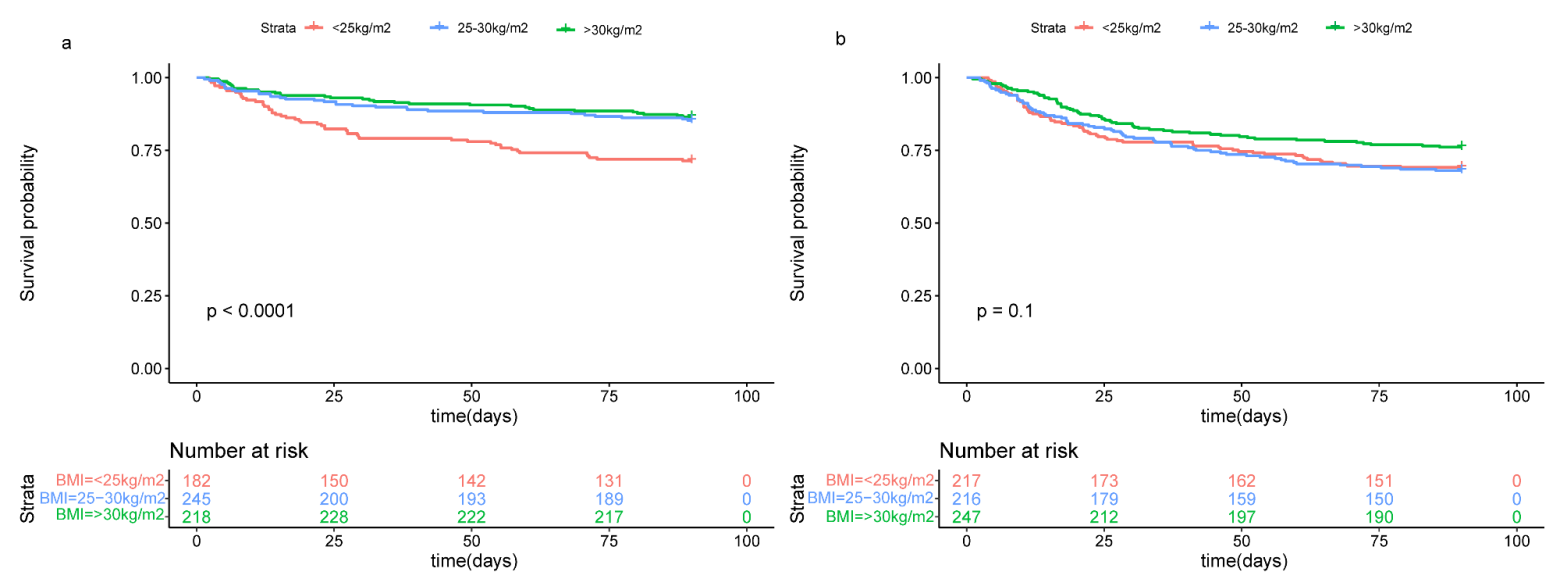
Kaplan-Meier survival curves for 90-day mortality for 1325 AHF patients in different groups (a) Kaplan-Meier survival curve of 90-day mortality in AHF patients with different BMI subgroups in High-SA group (b) Kaplan-Meier survival curve of 90-day mortality in AHF patients with different BMI subgroups in Low-SA group. AHF, acute heart failure; BMI, body mass index; SA, serum albumin

### Prognosis

**Fig. 2:**
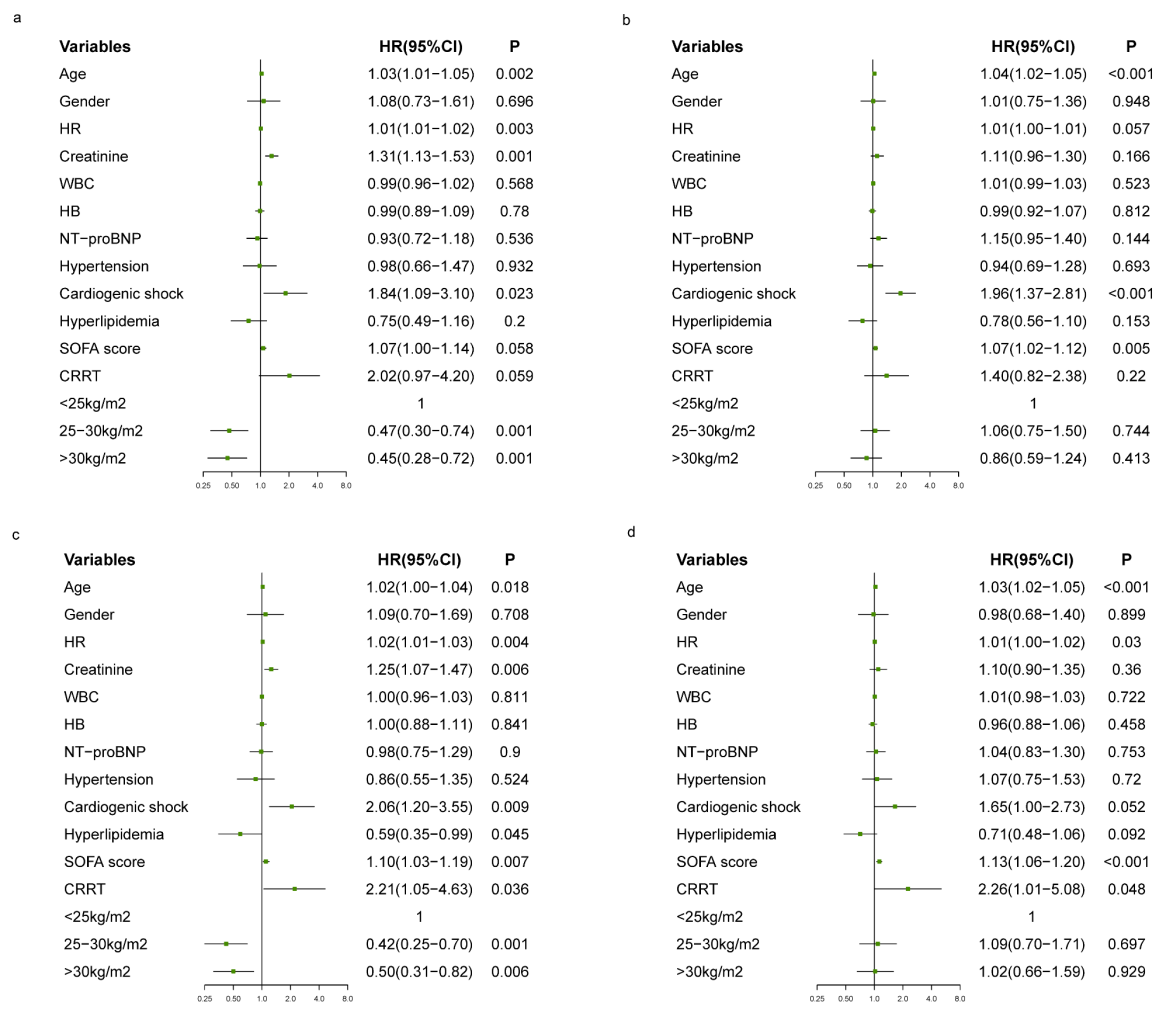
The Cox regression analysis of 90-day mortality for AHF patients in different groups before and after PSM (a) The Cox regression analysis of 90-day mortality for AHF patients with different BMI subgroups in High-SA group before PSM (b) The Cox regression analysis of 90-day mortality for AHF patients with different BMI subgroups in Low-SA group before PSM (c) The Cox regression analysis of 90-day mortality for AHF patients with different BMI subgroups in High-SA group after PSM (d) The Cox regression analysis of 90-day mortality for AHF patients with different BMI subgroups in Low-SA group after PSM. AHF, acute heart failure; BMI, body mass index; SA, serum albumin; HR, heart rate; WBC, white blood cell count; HB, Hemoglobin; NT-proBNP, N-terminal probrain natriuretic peptide; SOFA, sequential organ failure assessment; CRRT, continuous renal replacement therapy; PSM, propensity-score matching; HR, Hazard ratio; CI, Confidence interval

Under different SA groups, the endpoint events for each BMI subgroups before and after PSM are presented in Table 2. Before PSM, In the High-SA group, the short-term mortality of overweight or obese patients was lower than that of under/normal BMI patients (in-hospital mortality: 7.3% vs. 15.4%, 6.9% vs. 15.4%; 90-day mortality: 14.2% vs. 28.6%, 13.8% vs. 28.6%). Those trends were present between BMI subgroups in the Low-SA group, but no differences were observed. After PSM, these phenomena could still be seen. Unexpectedly, before and after PSM, whether hospital LOS or ICU LOS was similar between different subgroups (Table 2, all *P* > 0.05). The Kaplan-Meier curve showed that obese patients in the High-SA group had a survival advantage, and this advantage gradually decreased with the decrease of BMI (Fig. 1a). This phenomenon could also be seen in the Low-SA group (Fig. 1b), but there was no significant difference between the subgroups. Table 3 and Fig. 2 showed the results of multi-factor regression analysis before and after PSM. Before PSM, after adjusting for other confounding variables such as age, gender, HR, creatinine, WBC, Hemoglobin, NT-proBNP, hypertension, hyperlipidemia, cardiogenic shock, SOFA score and CRRT, age, HR, creatinine level, cardiogenic shock, overweight, and obesity were independent predictors of 90-day mortality in patients with SA ≥ 3.5 g/dL (Fig. 2a). Moreover, overweight and obesity were negatively correlated with 90-day mortality (HR 0.47, 95% CI 0.30–0.74; HR 0.45, 95%CI 0.28–0.72; Table 3). Although obese patients had a 14% lower risk of death in the Low-SA group, the association for survival advantage was attenuated and did not differ statistically between subgroups (Fig. 2b; Table 3). After PSM, after adjusting for covariates, in the High-SA group, the 90-day risk of death in overweight or obese patients was reduced by 50–58% (Fig. 2c; Table 3), while in the Low-SA group, overweight or obesity did not show obvious significant protective effect (hazard ratios [HR] 1.09, 95% confidence interval [CI] 0.70–1.71); HR 1.02, 95% CI 0.66 − 0.59) (Fig. 2d; Table 3).

**Table 2:** Under different SA groups, the endpoint outcomes for BMI subgroups before and after PSM

Outcome	High-SA group (BMI, kg/m^2^)			*p*-value	Low-SA group (BMI, kg/m^2^)			*p*-value
	< 25 kg/m^2^	25-30 kg/m^2^	> 30 kg/m^2^		< 25 kg/m^2^	25-30 kg/m^2^	> 30 kg/m^2^	
**Before PSM**								
Hospital mortality, %	15.4	7.3	6.9	0.005^**^	17.5	18.5	13.8	0.342
90-day mortality, %	28.6	14.2	13.8	< 0.001^***^	30.9	31.9	23.9	0.110
Hospital LOS, days	11(7–15)	11(7–16)	10(7–15)	0.502	12(9–20)	12(8–18)	13(9–21)	0.189
ICU LOS, days	4.2(3.0-11.1)	4.5(3.1-7.0)	4.1(2.9–6.8)	0.487	5.0(3.4–9.7)	5.0(3.4–8.5)	5.3(3.3–9.4)	0.931
**After PSM**								
Hospital mortality, %	16.1	6.6	8.7	0.011^*^	12.1	13.1	12.8	0.962
90-day mortality, %	28.2	13.1	15.8	0.001^**^	25.5	26.9	23.2	0.714
Hospital LOS, days	11(7–15)	11(7–17)	10(7–16)	0.565	12(9–18)	11(7–17)	13(8–20)	0.138
ICU LOS, days	4.1(7.1–2.9)	4.3(3.1–7.1)	4.1(3.0-7.1)	0.416	4.6(3.1–8.3)	4.9(3.2–7.5)	5.1(3.2–8.8)	0.683

BMI, body mass index; ICU, intensive care unit; LOS, length of stay; SA, serum albumin; PSM, propensity-score matching; ^*^P < 0.05; ^**^P < 0.01; ^***^P < 0.001

**Table 3:** Cox regression analysis of 90-day mortality in AHF patients grouped by SA and BMI

Subgroups			Unadjusted				Adjusted			
			HR		95%CI	*P*	HR		95%CI	*P*
**Before PSM**										
High-SA	< 25 kg/m^2^		Ref							
	25-30 kg/m^2^		0.46	0.29–0.72		0.001^**^	0.47	0.30–0.74		0.001^**^
	> 30 kg/m^2^		0.43	0.28–0.66		< 0.001^***^	0.45	0.28–0.72		0.001^**^
Low-SA	< 25 kg/m^2^		Ref							
	25-30 kg/m^2^		1.03	0.74–1.45		0.854	1.06	0.75–1.50		0.744
	> 30 kg/m^2^		0.73	0.52–1.04		0.080	0.86	0.59–1.24		0.413
**After PSM**										
High-SA	< 25 kg/m^2^		Ref							
	25-30 kg/m^2^		0.42	0.26–0.70		0.001^**^	0.42	0.25–0.70		0.001^**^
	> 30 kg/m^2^		0.52	0.33–0.82		0.005^**^	0.50	0.31–0.82		0.006^**^
Low-SA	< 25 kg/m^2^		Ref							
	25-30 kg/m^2^		1.06	0.69–1.63		0.800	1.09	0.70–1.71		0.697
	> 30 kg/m^2^		0.90	0.59–1.36		0.609	1.02	0.66–1.59		0.929

Adjusted for age, gender, heart rate, serum creatinine, white blood cell count (WBC), Hemoglobin, N-terminal probrain natriuretic peptide (NT-proBNP), hypertension, hyperlipidemia, cardiogenic shock, sequential organ failure assessment (SOFA) score and continuous renal replacement therapy (CRRT); SA, serum albumin; AHF, acute heart failure; BMI, body mass index; Ref, reference; HR, Hazard ratio; CI, Confidence interval; PSM, propensity-score matching; ^*^P < 0.05; ^**^P < 0.01; ^***^P < 0.001

### Sensitivity analyses

**Fig. 3:**
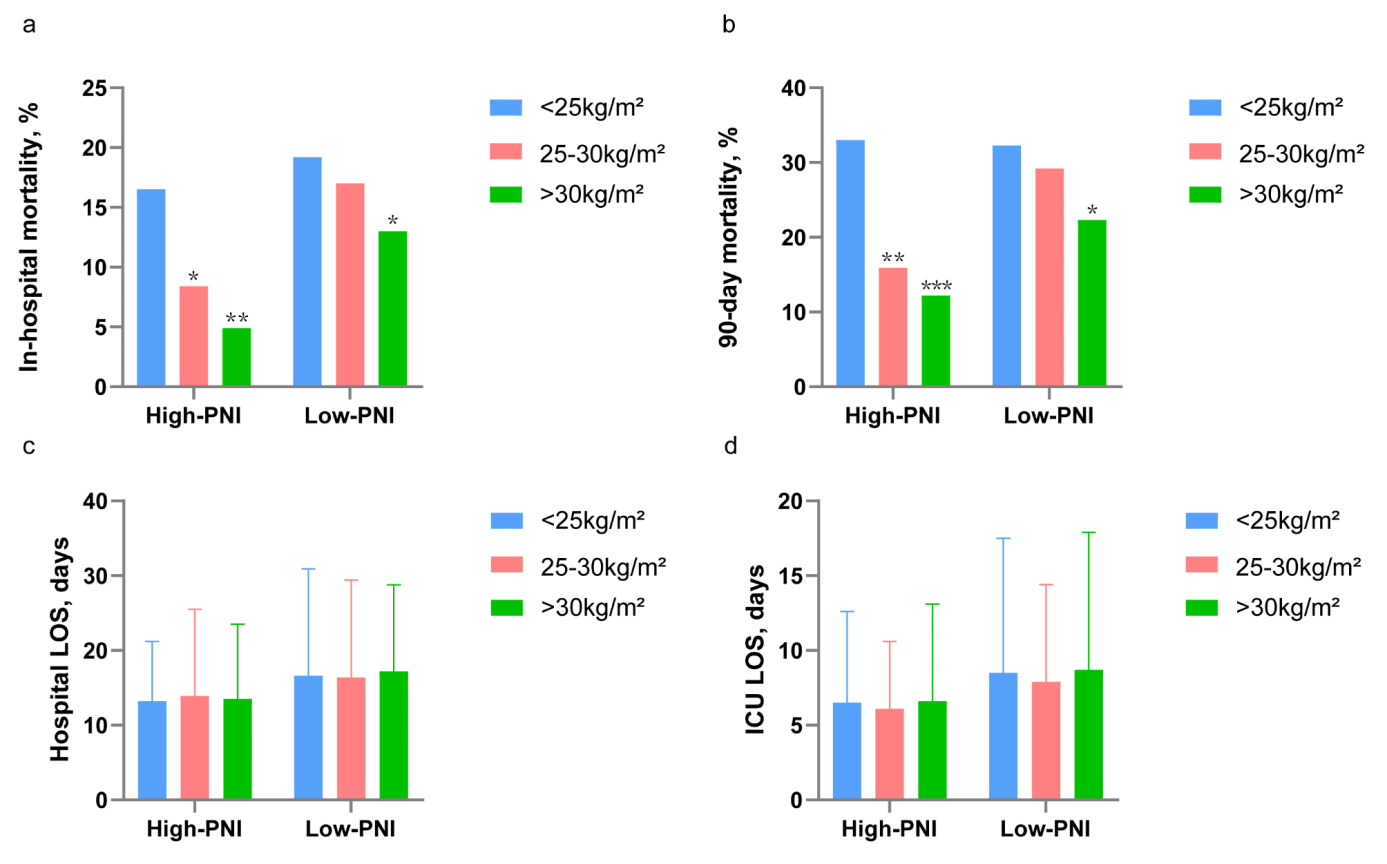
Clinical outcomes in different groups of AHF patients (a) In-hospital mortality of AHF patients in each BMI subgroup under different PNI groups (b) 90-day mortality of AHF patients in each BMI subgroup under different PNI groups (c) Hospital LOS of AHF patients in each BMI subgroup under different PNI groups (d) ICU LOS of AHF patients in each BMI subgroup under different PNI groups. AHF, acute heart failure; BMI, body mass index; ICU: Intensive Care Unit; LOS: length of stay; PNI, prognostic nutritional index; Compared with BMI < 25 kg/m2, * *P* < 0.05; **P < 0.01; ***P < 0.001

**Fig. 4:**
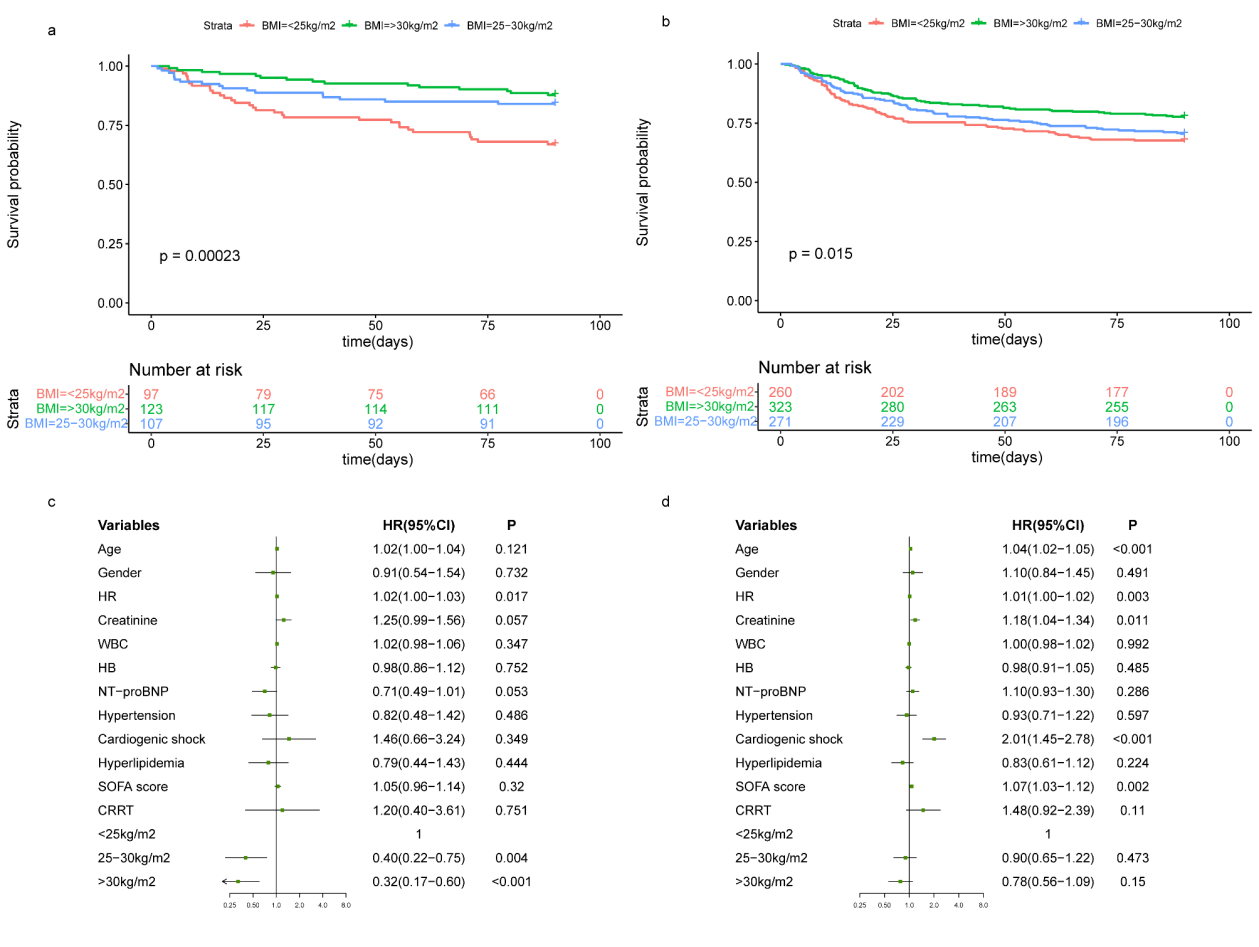
90-day survival analysis of different groups of AHF patients (a) Kaplan-Meier survival curve of 90-day mortality in AHF patients with different BMI subgroups in High-PNI group (b) Kaplan-Meier survival curve of 90-day mortality in AHF patients with different BMI subgroups in Low-PNI group (c) The Cox regression analysis of 90-day mortality for AHF patients with different BMI subgroups in High-PNI group (d) The Cox regression analysis of 90-day mortality for AHF patients with different BMI subgroups in Low-PNI group. AHF, acute heart failure; BMI, body mass index; HR, heart rate; WBC, white blood cell count; HB, Hemoglobin; NT-proBNP, N-terminal probrain natriuretic peptide; SOFA, sequential organ failure assessment; CRRT, continuous renal replacement therapy; HR, Hazard ratio; CI, Confidence interval

Furthermore, we also performed a subgroup analysis of 1181 patients with available PNI scores. the PNI was < 38 in 327(27.7%) and ≥ 38 in 854(72.3%), it could be seen that the clinical outcomes of the Low-PNI group were worse (Additional File Table 1). In the High-and Low-PNI groups, the in-hospital and 90-day mortality of overweight or obese patients were lower than those of patients with under/normal BMI (Fig. 3a and b), and the total LOS and ICU LOS were similar (Fig. 3c and d). The Kaplan-Meier curve showed that the cumulative survival rate of obese patients in the High-PNI group was the highest, and that of patients with under/normal BMI was the lowest (Fig. 4a). Whereas, this difference disappeared in the Low-PNI group (Fig. 4b). After adjusting for confounding factors, overweight and obesity were independently associated with better survival in the High-PNI group (Fig. 4c; Table 4), while the association was insignificant and did not make a significant difference in the Low-PNI group (Fig. 4d; Table 4).

**Table 4:** Cox regression analysis of 90-day mortality in AHF patients grouped by PNI and BMI

Subgroups		Unadjusted			Adjusted		
		HR	95%CI	*P*	HR	95%CI	*P*
High-PNI	< 25 kg/m^2^	Ref					
	25-30 kg/m^2^	0.45	0.25–0.81	0.007^**^	0.40	0.22–0.75	0.004^**^
	> 30 kg/m^2^	0.32	0.18–0.60	< 0.001^***^	0.32	0.17–0.60	< 0.001^***^
Low-PNI	< 25 kg/m^2^	Ref					
	25-30 kg/m^2^	0.87	0.64–1.18	0.360	0.90	0.65–1.22	0.473
	> 30 kg/m^2^	0.63	0.46–0.87	0.005^**^	0.78	0.56–1.09	0.150

Adjusted for age, gender, heart rate, serum creatinine, white blood cell count (WBC), Hemoglobin, N-terminal probrain natriuretic peptide (NT-proBNP), hypertension, hyperlipidemia, cardiogenic shock, sequential organ failure assessment (SOFA) score and continuous renal replacement therapy (CRRT);

AHF, acute heart failure; PNI, prognostic nutritional index; BMI, body mass index; Ref, reference; HR, Hazard ratio; CI, Confidence interval; ^*^P < 0.05; ^**^P < 0.01; ^***^P < 0.001

## Discussion

This paper was conducted to explore the effect of BMI and nutritional status on the short-term mortality of patients with AHF. We found that short-term mortality in the overweight or obese group was significantly lower than that in under/normal group in well-nourished patients. Surprisingly, this survival advantage was significantly attenuated or even eliminated in malnourished patients, suggesting that malnutrition may partially interfere with the association between obesity and mortality in with AHF.

Obesity, a manifestation of overnutrition, is a known risk factor for coronary heart disease and HF. While several studies have shown that Obesity, as measured by BMI and other indices, is associated with better survival in HF [7], some studies have strongly refuted this phenomenon [23]. In any case, the obesity paradox is at least not applicable in the general population or patients without cardiovascular disease, weight loss recommendations are still advocated, and further exploration and demonstration are needed in HF. In addition, malnutrition is very common in many diseases [16], such as AHF, and is significantly correlated with high mortality [13], long hospital stay time [24], and many complications [25]. The indicators that have been identified for monitoring nutritional status mainly include subjective overall assessment (SGA), PNI, nutritional risk screening (NRS-2002) and some biochemical indicators Such as total cholesterol, SA and total lymphocyte count, which could be used to predict the prognosis of patients with HF [26]. Considering that SA, height, weight, and lymphocyte counts are easy to obtain clinically and can reflect immune, inflammatory, and metabolic conditions, we adopt SA, PNI as indicators to assess nutritional status and also found that malnourished patients had higher levels of white blood cells, higher mortality, and longer hospital stays, consistent with previous studies [27]. Previous studies mostly paid attention to exploring the effects of Obesity or single nutritional indicators on cardiovascular outcomes. A study on the combined effect of Obesity and nutrition in critically ill patients showed that obese and malnourished patients had poorer prognoses than obese and well-nourished patients [28]. Moreover, the impact of this comprehensive factor on the prognosis of AHF remains to be explored. Therefore, this study explored the effect of BMI combined with nutritional indicators on the prognosis of patients with AHF and provided a basis for clinicians to identify risk factors as soon as possible.

Numerous clinical studies have confirmed that Obesity is beneficial to the prognosis of patients diagnosed with HF, which might be related to a variety of factors such as high energy reserves [29], lower circulating levels of B-type natriuretic peptide [30], neuroendocrine protection [31], higher blood volume and genetic factors, etc. Studies have also shown that compared with HF patients with high BMI, patients with relatively normal BMI may have relatively low muscle mass and muscle strength, also known as sarcopenia, which would lead to poor cardiopulmonary function and adverse clinical prognosis in patients with HF [32]. Of note, most of the studies supporting the “obesity paradox” comes from retrospective epidemiological studies, and it is necessary to be alert to potential key confounding factors or collider biases that contributed to this paradox. The role of confounding factors has been explored in different environments. For example, the “obesity paradox” did not exist in chronic ischemic HF [33], and HF patients with diabetes could not benefit from the obesity paradox [34], or no significant correlation was found between higher BMI and lower mortality in female patients with HF [35]. The obesity paradox is still controversial in HF with different phenotypes or etiologies, and since we did not have access to information on Left ventricular ejection fraction and the etiology of HF, the conclusion needed to be interpreted with caution and further studied.

Under different nutritional statuses, we found that in well-nourished (SA ≥ 3.5 g/dL, or PNI ≥ 38) patients, overweight or obesity showed lower in-hospital and 90-day crude mortality, and under/normal BMI had the worst prognosis. Unexpectedly, in malnourished (SA < 3.5 g/dL, or PNI < 38) patients, there was no significant difference in the in-hospital crude mortality between BMI subgroups, while 90-day mortality decreased with increasing BMI. After multivariate adjustment, we found that overweight and obesity were negatively correlated with the 90-day all-cause mortality of well-nourished patients, suggesting that the obesity paradox may exist. Unfortunately, no statistical difference was found in malnourished patients with AHF despite a 14-22% lower 90-day mortality risk with obese BMI compared with under/normal BMI. One explanation was that malnutrition could promote pulmonary edema, fluid retention, diuretic resistance, oxidative stress, and inflammatory conditions, making heart failure symptoms progressively worse, leading to poor clinical prognosis. It might partially offset (cover up) the protective effect of Obesity. Another explanation is that unmeasured confounding variables influenced our estimates of reported associations. Therefore, this finding should be replicated in a larger population before its implications for public health are fully appreciated. In addition, we unexpectedly found that total LOS and ICU LOS were similar among the groups, which was inconsistent with previous results and might be limited by sample size.

Several limitations in the present study should be put forward. Firstly, this was a single-center retrospective study, which could only infer an association, not a causal relationship. Furthermore, despite the use of sophisticated statistical analysis strategies (sensitivity analysis, PSM), the effect of potential selection bias and confounding bias could not be completely eliminated. For example, our AHF patients were sampled from ICU patients only and those patients might be more severe and need better care compared to the general AHF patients leading to potentially overestimate the association, and some variables related to adverse clinical outcomes were missing to some extent. Therefore, caution is needed in interpreting this conclusion. Secondly, we used the first BMI data during hospitalization without the follow-up of its fluctuations, there might be liquid retention. Therefore, we could not ignore the fact that BMI might be overestimated in some patients. Finally, we adopted SA and PNI as substitute indicators of nutritional status rather than a set of standardized diagnostic features recommended by the Society of Nutrition and Dietetics to define malnutrition, and there might be some deviations. Therefore, large-scale randomized controlled trials may be needed in the future to provide more definitive evidence for this conclusion.

## Conclusion

In conclusion, among diagnosed AHF patients, overweight or obesity might have a protective effect on short-term mortality, especially in well-nourished patients. While in malnourished patients, the survival advantage of overweight or obesity was relatively lower, for these patients, it may be necessary to early nutritional assessment and intervention therapy. However, further exploration and validation of nutritional indicators combined with BMI in a larger population is needed to guide clinicians on the best strategy for treatment.

## Electronic supplementary material

Below is the link to the electronic supplementary material.

Additional File Figure 1: Flow chart of screening patients. AHF, acute heart failure; SA, serum albumin; ACS, acute coronary syndrome.

Additional File Figure 2: Distribution of patients in High- and Low-SA groups after PSM. PSM, propensity-score matching; SA, serum albumin.

Additional File Table 1: Baseline characteristics of the study population grouped by PNI

## Data Availability

The datasets used and analyzed during the current study are available from the corresponding author on reasonable request. The website of MIMIC database: https://mimic.mit.edu/.

## Abbreviations

HF: Heart failure
AHF: Acute heart failure
BMI: Body mass index
SA: Serum albumin
PNI: Prognostic nutritional index
MIMIC-III: Medical Information Mart for Intensive Care III
ICU: Intensive Care Unit
ICD: International Classification of Diseases
LOS: Length of stay
SOFA: Sequential Organ Failure Assessment
CRRT: Continuous renal replacement therapy
ACEI: Angiotensin converting enzyme inhibitor
AF: Atrial fibrillation
ACS: Acute coronary syndrome
COPD: Chronic obstructive pulmonary disease
WBC: White blood cell
HB: Hemoglobin
AG: Anion gap
NT-proBNP: N-terminal probrain natriuretic peptide
MI: Myocardial infarction
SD: Standard deviation
IQR: Interquartile range
HR: Hazard ratios
PSM: Propensity-score matching
CI: Confidence interval
KM: Kaplan-Meier
