# Supplementary figures and images for: Effects of obesity on short-term mortality in patients with acute heart failure under different nutritional status
Source: BMC Cardiovasc Disord. 2023 Apr 29;23:221. doi: 10.1186/s12872-023-03206-x (PMC10149014; doi:10.1186/s12872-023-03206-x)

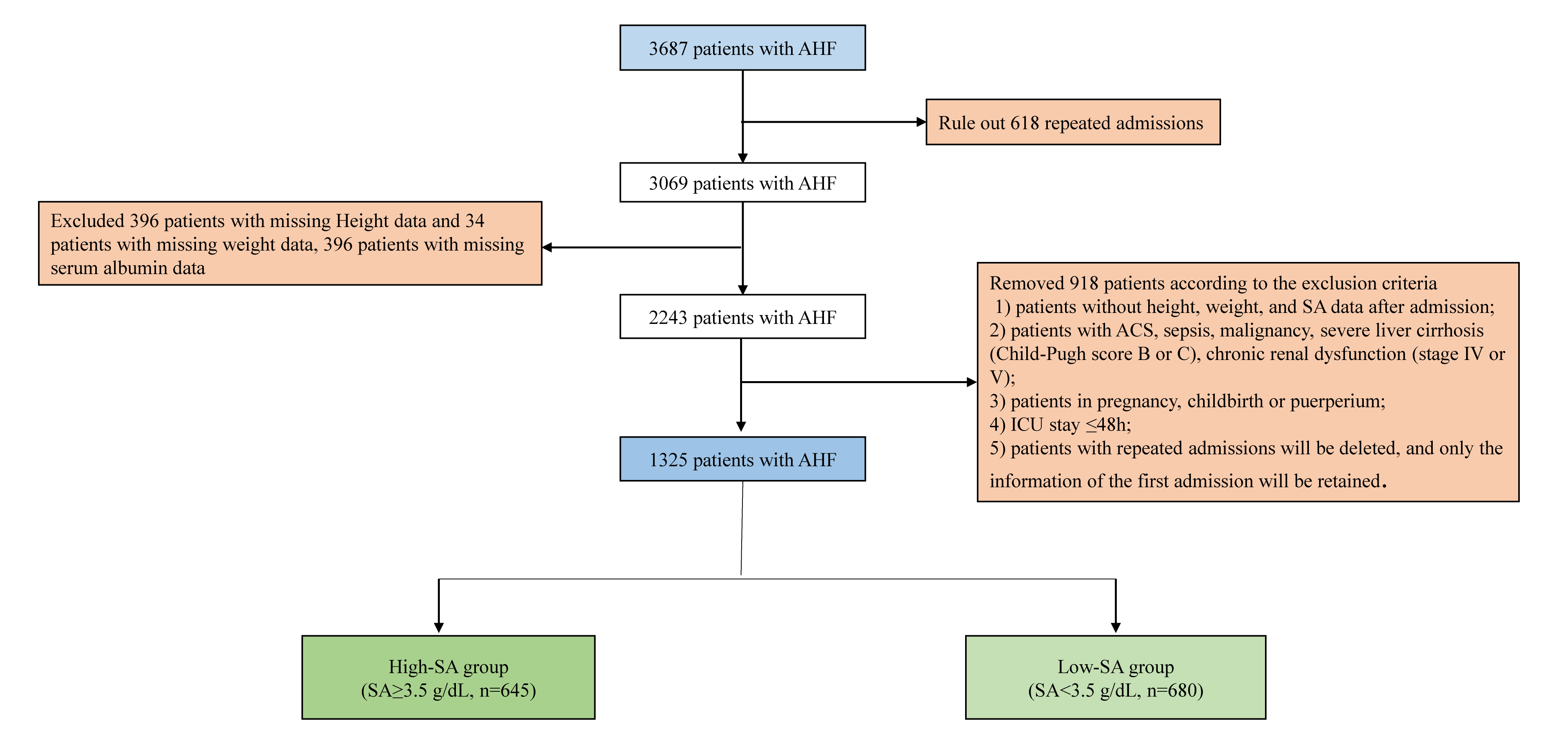

Supplement: Supplementary file 1 — Additional File Figure 1: Flow chart of screening patients. AHF, acute heart failure; SA, serum albumin; ACS, acute coronary syndrome. [file 12872_2023_3206_MOESM1_ESM.png]

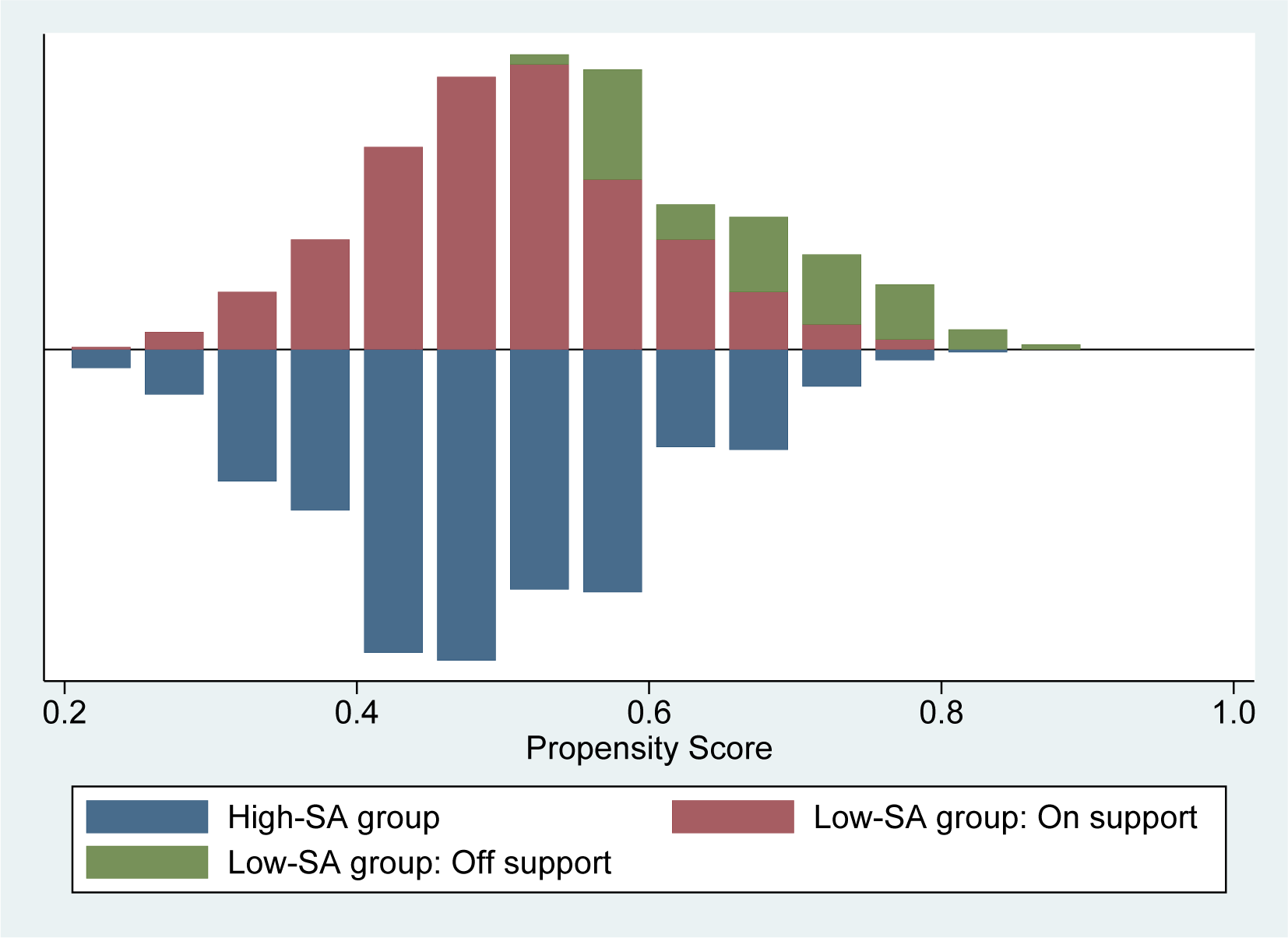

Supplement: Supplementary file 2 — Additional File Figure 2: Distribution of patients in High- and Low-SA groups after PSM. PSM, propensity-score matching; SA, serum albumin. [file 12872_2023_3206_MOESM2_ESM.png]
