# Supplementary material for: Effects of obesity on short-term mortality in patients with acute heart failure under different nutritional status
Source: BMC Cardiovasc Disord. 2023 Apr 29;23:221. doi: 10.1186/s12872-023-03206-x (PMC10149014; doi:10.1186/s12872-023-03206-x)
Supplement: Supplementary file 3 — Additional File Table 1: Baseline characteristics of the study population grouped by PNI [file 12872_2023_3206_MOESM3_ESM.docx]

Supplemental Table 1 Baseline characteristics of the study population grouped by PNI

| variable | Total  (n=1181) | High-PNI  (≥38, n=327) | Low-PNI  (<38, n=854) | | *P*-value |
| --- | --- | --- | --- | --- | --- |
| Demographics |  | | | | |
| Age(years) | 72.7±13.0 | 72.0±12.7 | 72.9±13.2 | | 0.316 |
| Male, n (%) | 595(50.4) | 173(52.9) | 422(49.4) | | 0.283 |
| Smoking, n (%) | 316(26.8) | 93(28.4) | 223(26.1) | | 0.419 |
| Weight (kg) | 79.4(65.9-95.3) | 80.3(66.9-95.9) | 79.0(65.5-95.0) | | 0.332 |
| BMI (kg/m^2^) | 28.0(24.1-32.8) | 28.3(24.4-33.3) | 27.9(23.8-32.8) | | 0.582 |
| HR (b.p.m) | 88(76-103) | 87(74 -101) | 89(77-104) | | 0.027^*^ |
| Hypertension, n (%) | 509(43.1) | 162(49.5) | 347(40.6) | | 0.006^**^ |
| Prior MI, n (%) | 152(12.9) | 42(12.8) | 110(12.9) | | 0.987 |
| Hyperlipidemia, n (%) | 345(29.2) | 105(32.1) | 240(28.1) | | 0.175 |
| COPD, n (%) | 85(7.2) | 26(8.0) | 59(6.9) | | 0.535 |
| AF, n (%) | 629(53.3) | 182(55.7) | 447(52.3) | | 0.307 |
| Diabetes, n (%) | 488(41.3) | 132(40.4) | 356(41.7) | | 0.680 |
| Cardiogenic shock, n (%) | 150(12.7) | 30(9.2) | 120(14.1) | | 0.024^*^ |
| Digoxin, n (%) | 189(16.0) | 53(16.2) | 136(15.9) | | 0.906 |
| β-blockers, n (%) | 968(82.0) | 272(83.2) | 696(81.5) | | 0.501 |
| ACEI, n (%) | 476(40.3) | 149(45.6) | 327(38.3) | | 0.023^*^ |
| Diuretic, n (%) | 1100(93.1) | 303(92.7) | 797(93.3) | | 0.686 |
| Statin, n (%) | 347(29.4) | 100(30.6) | 247(28.9) | | 0.576 |
| WBC, 10^9^/L | 11.0(8.1-15.1) | 11.1(7.7-15.0) | 10.9(8.1-15.1) | | 0.720 |
| Lymphocytes, 10^9^/L | 1.06(0.67-1.61) | 1.22(0.74-1.99) | 1.05(0.64-1.49) | | <0.001^***^ |
| HB, mg/dL | 10.1(8.9-11.8) | 10.4(9.0-12.3) | 10.0(8.9-11.6) | | 0.011^*^ |
| AG, mmol/L | 14(12-17) | 14(12-17) | 14(12-17) | | 0.235 |
| Creatinine, mg/dL | 1.2(0.9-1.9) | 1.1(0.9-1.6) | 1.3(0.9-2.0) | | 0.001^**^ |
| SA, g/dL | 3.4(3.0-3.8) | 4.0(3.8-4.2) | 3.2(2.8-3.5) | | <0.001^***^ |
| Glucose, mg/dL | 123(101-156) | 124(103-154) | 123(99-157) | | 0.325 |
| NT-proBNP, n (%) |  | | | 0.180 | |
| <6217 pg/mL | 279(23.6) | 89(27.2) | 190(22.2) | | |
| ≥6217pg/mL | 241(20.4) | 61(18.7) | 180(21.1) | | |
| No test | 661(56.0) | 177(54.1) | 484(56.7) | | |
| SOFA score | 5.0(3.0-7.0) | 4.0(3.0-7.0) | 5.0(3.0-7.0) | | 0.014^*^ |
| CRRT, n (%) | 62(5.2) | 13(4.0) | 49(5.7) | | 0.003^**^ |
| Assisted ventilation, n (%) | 654(55.4) | 174(53.2) | 480(56.2) | | 0.354 |
| Hospital mortality, n (%) | 169(14.3) | 31(9.5) | 138(16.2) | | 0.003^**^ |
| 90-day mortality, n (%) | 299(25.3) | 64(19.6) | 235(27.5) | | 0.005^**^ |
| Hospital LOS (days) | 11.9(7.8-18.0) | 10.7(7.3-16.1) | 12.5(8.0-18.7) | | 0.009^**^ |
| ICU LOS (days) | 4.9(3.2-8.4) | 4.1(3.0-7.6) | 5.1(3.3-8.8) | | <0.001^***^ |

PNI, prognostic nutritional index; BMI, body mass index; HR, heart rate; MI, myocardial infarction; AF, Atrial fibrillation; COPD, chronic obstructive pulmonary disease; ACEI, angiotensin-converting enzyme inhibitors; WBC, white blood cell count; HB, Hemoglobin; SA, serum albumin; AG, anion gap; NT-proBNP, N‐terminal probrain natriuretic peptide; SOFA, sequential organ failure assessment; CRRT, continuous renal replacement therapy; ICU, Intensive Care Unit; LOS, length of stay; ^*^*P*<0.05; ^**^*P*<0.01; ^***^*P*<0.001.
